# Development of an Experimental Method Using a Portable Photosynthesis-Monitoring System to Measure Respiration Rates in Small-Sized Insects

**DOI:** 10.3390/insects16060616

**Published:** 2025-06-10

**Authors:** Bi-Yue Ding, Qin-Qin Xu, Yu-Jing Liu, Yu-Hong Zhong, Yan Zhou

**Affiliations:** 1 Instrument and Equipment Sharing Service Center, Academy of Agricultural Sciences, Southwest University, Chongqing 400715, China; zhouyanswu@126.com; 2 Key Laboratory of Entomology and Pest Control Engineering, College of Plant Protection, Southwest University, Chongqing 400715, China; qinqinxu999@163.com (Q.-Q.X.); yjingliu1@163.com (Y.-J.L.); zyz123579@email.swu.edu.cn (Y.-H.Z.)

**Keywords:** respiration rates, photosynthesis-monitoring system, insects, various treatments, RNAi

## Abstract

Respiration rates in insects are critical for survival and environmental adaptation, being influenced by developmental stages, environmental conditions, and the regulation of mitochondrial protein-coding genes. However, methods for field-based measurements in small-sized insects remain limited. In this study, we established a portable photosynthesis system to quantify respiration rates in five small-sized insects (body length < 8 mm): *Acyrthosiphon pisum*, *Aphis citricidus*, *Tuta absoluta*, *Tribolium castaneum*, and *Bactrocera dorsalis*. We tested its effectiveness across life stages and under diverse treatments, including light/dark cycles, insecticides, temperature shifts, starvation, mitochondrial inhibitors, and RNA interference. The system exhibited high sensitivity and reproducibility rates, revealing stage-specific respiration patterns. Various treatments, as well as expression changes in mitochondrial protein-coding genes, significantly affected respiration rates. This study validates the portable system as a reliable tool for insect respiration studies and highlights regulatory networks associated with respiratory plasticity. These findings enhance experimental methodologies and advance our understanding of insect adaptation to environmental stressors and pest control strategies.

## Simple Summary

Understanding how insects breathe is essential for both ecological research and pest control. However, accurately measuring the respiration rates of small-sized insects has been challenging due to a lack of suitable tools. This study evaluated the use of a portable photosynthesis system, originally developed for plants, to measure insect respiration. The results demonstrated that this method is effective across various insect species and developmental stages. Moreover, factors such as temperature, starvation, and specific chemicals significantly influenced respiration rates. Silencing a mitochondrial protein-coding gene through RNA interference reduced respiration and increased resistance to certain stresses. Overall, this study introduces a novel experimental method for measuring insect respiration, offering insights into their adaptation mechanisms and providing a potential tool for improved pest management. These findings also contribute valuable information to our understanding of how insects interact with their environment and respond to challenges such as climate change.

## 1. Introduction

The respiratory system in insects is essential for environmental adaptation, development, and survival [1,2]. To meet their high metabolic demands, insects depend on efficient gas exchange [3]. Respiration rates are influenced by multiple factors, including body size, activity level, food availability, temperature, and oxygen concentration [4,5]. Activities such as flight or mating can substantially increase respiration rates [6]. Temperature has a direct effect on insect respiration by influencing the metabolic rate, thereby affecting thermoregulation and respiratory efficiency under changing conditions [7]. These mechanisms underpin insects’ adaptability to environmental fluctuations, particularly in the context of global climate change [8,9]. Understanding insect respiration, therefore, holds ecological relevance and practical value in pest management [3,10]. For instance, strategies that target respiratory processes have led to the development of effective pesticides and control methods [11,12,13,14]. The respiration rate is thus a key parameter in studies of insect biology, ecology, and environmental interaction [4,15]. However, current techniques for measuring respiration in small-sized insects (body length < 8 mm) are limited by low sensitivity and poor field operability [16,17].

The portable photosynthesis system has become a widely used tool for quantifying photosynthetic parameters across diverse plant species and environmental conditions owing to its ease of use, standardized protocols, and high degree of experimental reproducibility [18,19,20,21,22]. Applications include studies in rapeseed [20], soybean [23], paper mulberry [24], cotton [25], mulberry [19], poplar [26], and switchgrass [27]. For example, it has been employed to measure the net photosynthesis, transpiration, intracellular CO_2_ concentration, and stomatal conductance in a *Brassica napus* variety resistant to Sclerotinia [20], as well as to assess the net photosynthetic rate in *Pakchoi* after seedling treatment with a polymeric hydrogel [28]. This system calculates the photosynthetic efficiency by simultaneously monitoring the CO_2_/H_2_O gas exchange, light intensity, and leaf temperature via integrated infrared sensors and environmental probes, enabling the precise quantification of photosynthesis and transpiration in field settings [29]. Based on these operational principles, the portable photosynthesis system offers untapped potential for measuring respiration in micro-insects by leveraging its high sensitivity to the CO_2_ flux, originally intended for detecting plant stomatal dynamics.

Mitochondria are essential organelles that regulate cellular respiration and energy production in eukaryotes [30]. In insects, mitochondrial genomes and associated genes play critical roles in respiratory function. Complete mitochondrial genome sequences have been reported for several species, including *Leucinodes orbonalis* [31], *Rhyzopertha dominica* [32], *Choroterpes yixingensis* [33], and *Aphis citricidus* [34], revealing 13 mitochondrial protein-coding genes. Although the expression patterns of these genes have been studied under various experimental conditions in some insect species [35,36,37], the relationship between their expression and respiration rates remains poorly understood in *Acyrthosiphon pisum*.

In this study, we developed a technique to measure respiration in small-sized insects, using a portable photosynthesis system. To validate its applicability, we assessed respiration rates across different life stages in multiple insect species and under various experimental conditions in *Ac. pisum*. This work presents a novel methodology for investigating insect respiration and contributes to a deeper understanding of insect adaptation mechanisms.

## 2. Materials and Methods

### 2.1. Insects

*Ac. pisum* was reared on *Vicia faba* seedlings, *Aphis citricidus* on *Citrus sinensis*, *Tuta absoluta* on *Solanum lycopersicum*, and *Bactrocera dorsalis* on an artificial diet, as previously described [38]. All the insects were maintained at 25 °C and 75% relative humidity and under a photoperiod of 14 h of light and 10 h of darkness. *Tribolium castaneum* was reared on a mixture of wheat flour and brewer’s yeast powder (in a 10:1 ratio) under conditions of 30 °C, 30% relative humidity, and a photoperiod cycle of 16 h of light and 8 h of darkness.

### 2.2. Sample Collection from Various Developmental Stages and Treatments

**Developmental stages.** Insects from different developmental stages of *Ac. pisum* and *Ap. citricidus* (first-instar nymphs (N1), second-instar nymphs (N2), third-instar nymphs, fourth-instar nymphs (N3), and three-day-old adults (AD)), *Ap. citricidus* (first-instar nymphs (N1), second-instar nymphs (N2), third-instar nymphs, fourth-instar nymphs (N3), three-day-old wingless adults (AD-WL), and three-day-old winged adults (AD-WL)), *T. absoluta* (eggs (E), larvae (L), pupae (P), and adults (AD)), *B. dorsalis* (eggs (E), first-instar larvae (L1), second-instar larvae (L2), third-instar larvae (L3), pupae (P), female adults (AD-F), and male adults (AD-M)), and *T. castaneum* (eggs (E), larvae (L), pupae (P), female adults (AD-F), and male adults (AD-M)) were collected for respiration rate measurements. For egg stages, 50 individuals were pooled per group; for larvae, pupae, and adults, 10 individuals were pooled. Each treatment included eight biological replicates.

**Photoperiod.** *Ac. pisum* individuals, maintained under a 14:10 h light–dark cycle, were sampled at two-hour intervals to assess respiration rate variations throughout the photoperiod. Ten aphids were pooled per group, with eight biological replicates.

**Temperatures.** *Ac. pisum* was exposed to acute thermal stress at 35 °C and 4 °C for 4, 8, or 12 h. Respiratory measurements were taken immediately after treatment. Aphids reared at 25 °C served as controls. Ten individuals were pooled per group, with eight biological replicates.

**Insecticides.** *Ac. pisum* was treated with LC_20_ concentrations of avermectin, β-cypermethrin, and imidacloprid [39]. Aphids treated with acetone were used as controls. Ten individuals were pooled per group, with eight biological replicates.

**Starvation.** *Ac. pisum* was starved for 4, 8, or 12 h. After each starvation period, individuals were collected for respiration rate measurements. Normally fed aphids served as controls. Ten insects were pooled per group, with eight biological replicates.

**Mitochondrial inhibitors.** *Ac. pisum* was treated with mitochondrial inhibitors (rotenone and antimycin A). Aphids treated with acetone were used as controls. Ten insects were pooled per group, with eight biological replicates.

### 2.3. Measured Respiration Rates

**Figure 1:**
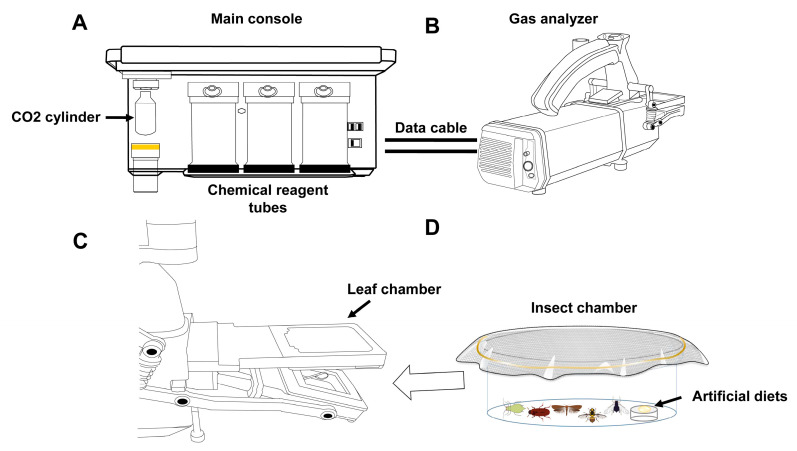
Device for measuring respiration rates of small-sized insects, using the portable photosynthesis system. (**A**) Main console. (**B**) Gas analyzer. (**C**) Position of the leaf chamber on the gas analyzer. (**D**) Insect chamber, equipped with an artificial-diet provision system. The insect chamber is inserted into the leaf chamber for gas-exchange measurements when containing experimental insects.

Insect respiration rates were measured using a modified LI-6800 photosynthesis system (Ecotek Technology, Beijing, China), coupled with a custom insect chamber containing a ventilated lid and an artificial feeding device (Figure 1), according to the manufacturer’s instructions with slight modifications. Briefly, after assembling the system and performing preheating checks and zeroing calibration through the instrument’s startup protocol, we configured the operational parameters as follows: a 500 μmol s^−1^ airflow, a 25 °C temperature control, and 400 μmol mol^−1^ CO_2_ levels with active scrubbing. The humidity was maintained between 50 and 75% using the system’s water vapor subsystem. The insects were placed in the transparent chamber, which was sealed within the LI-6800 leaf chamber (Ecotek Technology, Beijing, China) and measurements were taken continuously for 10 min, with automatic data collection every 2 min. The insects were given time to reach a stable respiratory state (ΔCO_2_ remained within 0.01 μmol mol^−1^). Respiration rates were calculated as CO_2_ emissions per minute, normalized to the insect’s body mass (μmol g^−1^ min^−1^), reflecting gas-exchange dynamics under controlled environmental conditions.

### 2.4. RT-qPCR

The primers for 13 mitochondrial protein-coding genes are listed in Table S1. The total RNA was extracted from treated samples using TRIzol reagent (Invitrogen, Carlsbad, CA, USA), and cDNA was synthesized using the PrimeScript RT reagent kit (Takara, Dalian, China). Quantitative PCR (qPCR) analysis was performed with NovoStart SYBR qPCR SuperMix (Novoprotein, Shanghai, China) to quantify target gene expression levels. Standard curves were generated with serial cDNA dilutions to determine the amplification efficiency and CT values. *Apactin* and *ApNADH* were used as reference genes for normalization via qBASE+ [40,41].

### 2.5. RNA Interference Assay

Double-stranded RNA (dsRNA) was synthesized using the TranscriptAid T7 high-yield kit (Thermo Scientific, Wilmington, DE, USA). Aphids were topically treated with 500 ng/μL dsRNA, with dsGFP as a control. The treated aphids were fed artificial diets for 12 h before respiration rate measurements (Section 2.3). Each group contained ten individuals, with eight replicates.

### 2.6. Statistical Analyses

Statistical analyses were performed using GraphPad Prism 7.0 (GraphPad Software, San Diego, CA, USA). Multiple group comparisons, including respiration rates among different developmental stages and various treatments and transcriptional patterns of mitochondrial protein-coding genes during light/dark periods and across developmental stages, were analyzed using one-way ANOVA with LSD post hoc tests (*p* < 0.05). Pairwise comparisons, including transcriptional patterns of mitochondrial protein-coding genes in different treatments and the RNAi assay, were assessed using Student’s *t*-test (*, *p* < 0.05; **, *p* < 0.01; ***, *p* < 0.001).

## 3. Results

### 3.1. Measurement of Respiration Rates in Different Insects, Using a Portable Photosynthesis-Monitoring System

**Figure 2:**
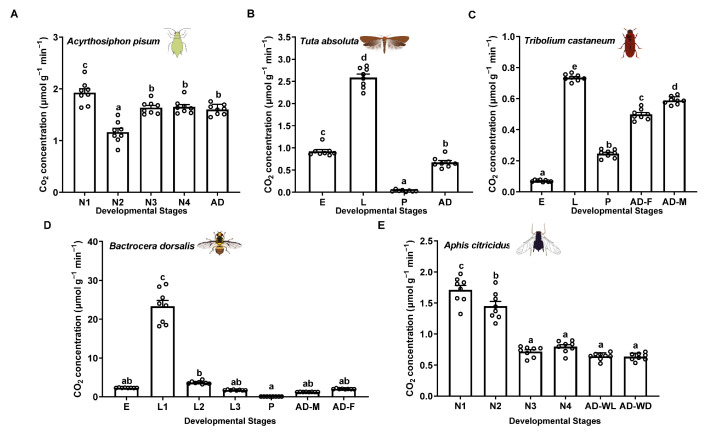
Respiration rates of different insects, using the portable photosynthesis-monitoring system. (**A**) Acyrthosiphon pisum. (**B**) Tuta absoluta. (**C**) Tribolium castaneum. (**D**) Bactrocera dorsalis. (**E**) Aphis citricidus. Values represent the mean ± standard error (SE) of eight biological replicates. Lowercase letters denote statistically significant differences determined through one-way ANOVA with LSD post hoc testing (*p* < 0.05).

To test the applicability of this method for various small-sized insects, we used a portable photosynthesis-monitoring system to measure the respiration rates of five insect species: *Ac. pisum* and *Ap. citricidus* (Hemiptera: Aphididae), *Tu. absoluta* (Lepidoptera: Gelechiidae), Tr. castaneum (Coleoptera: Tenebrionidae), and B. dorsalis (Diptera: Tephritidae) across different developmental stages or morphs. The results demonstrate that this method offers high sensitivity, ease of use, and excellent reproducibility. The respiration rate profiles revealed that second-instar nymphs exhibited the lowest respiration rates (1.16 μmol g^−1^ min^−1^), while first-instar nymphs showed the highest (1.93 μmol g^−1^ min^−1^) in *Ac. pisum* (Figure 2A). In *Tu. absoluta*, pupae had the lowest rates (0.03 μmol g^−1^ min^−1^), whereas larvae displayed the highest rates (2.58 μmol g^−1^ min^−1^) (Figure 2B). In *Tr. Castaneum*, eggs had the lowest respiration rates (0.07 μmol g^−1^ min^−1^), while larvae exhibited the highest (0.74 μmol g^−1^ min^−1^) (Figure 2C). In B. dorsalis, pupae displayed the lowest rates (0.06 μmol g^−1^ min^−1^), while first-instar larvae exhibited the highest (23.31 μmol g^−1^ min^−1^) (Figure 2D). In Ap. citricidus, winged adults (0.63 μmol g^−1^ min^−1^), wingless adults (0.64 μmol g^−1^ min^−1^), third-instar nymphs (0.72 μmol g^−1^ min^−1^), and fourth-instar nymphs (0.79 μmol g^−1^ min^−1^) had the lowest respiration rates, while first-instar nymphs showed the highest (1.71 μmol g^−1^ min^−1^) (Figure 2E).

### 3.2. Measurement of Respiration Rates of Ac. pisum Under Various Treatments, Using a Portable Photosynthesis-Monitoring System

**Figure 3:**
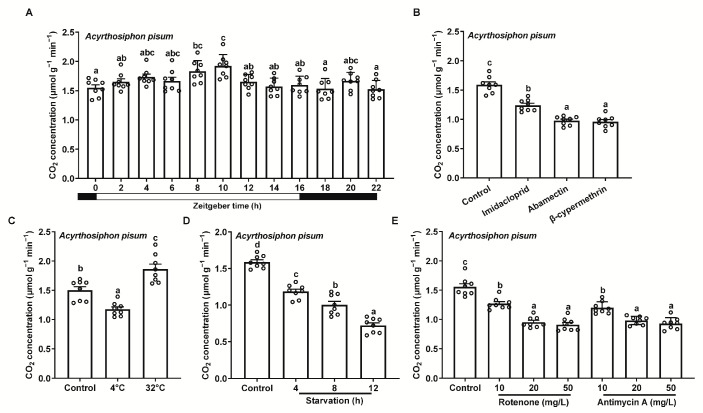
Respiration rates of Acyrthosiphon pisum under different treatments, using the portable photosynthesis-monitoring system. (**A**) Light/dark cycles over a full photoperiod. (**B**) Insecticide exposure. (**C**) Varying temperatures. (**D**) Starvation at various time points. (**E**) Mitochondrial inhibitors. Values represent the mean ± standard error (SE) of eight biological replicates. Lowercase letters denote statistically significant differences determined through one-way ANOVA with LSD post hoc testing (*p* < 0.05).

To assess the applicability of this method for measuring insect respiration under different conditions, we tested *Ac. pisum* exposed to light/dark cycles over a full photoperiod, insecticides, varying temperatures, starvation periods, and mitochondrial inhibitors at different concentrations. The respiration rates remained stable throughout the photoperiod, with only a slight initial increase followed by a subsequent decrease during light periods (Figure 3A). All three insecticides (imidacloprid, abamectin, and β-cypermethrin) reduced respiration rates (Figure 3B). High temperatures (32 °C) significantly increased the respiration rates, whereas low temperatures (4 °C) markedly decreased them (Figure 3C). Starvation also reduced respiration rates, with the reduction intensifying over time (Figure 3D). Both mitochondrial inhibitors (rotenone and antimycin A) suppressed respiration rates, with progressive decreases at higher concentrations (Figure 3E).

### 3.3. Measurement of Respiration Rates of Ac. pisum Under the Silencing of a Key Mitochondrial Protein-Coding Genes, Using a Portable Photosynthesis-Monitoring System

To measure the respiration rates of Ac. pisum under the silencing of a key mitochondrial protein-coding gene, using the portable photosynthesis-monitoring system, we systematically quantified the expression profiles of 13 protein-coding genes. The expression profiles showed stable expression levels during light periods, with lower expression levels later in the light phase and dynamic changes during darkness (zeitgeber times ZT16–ZT22), peaking at ZT18 or ZT22 (Figure S1). Developmental-stage-specific profiles revealed higher gene expression levels in younger nymphs. For example, NAD1, NAD4, NAD5, Cytb, COX1, COX2, COX3, and ATP6 were highly expressed in second-instar nymphs, while NAD2, NAD4L, NAD6, and ATP8 peaked in third-instar nymphs (Figure S2). Larger insects (fourth-instar nymphs and adults) generally showed lower expression levels.

**Figure 4:**
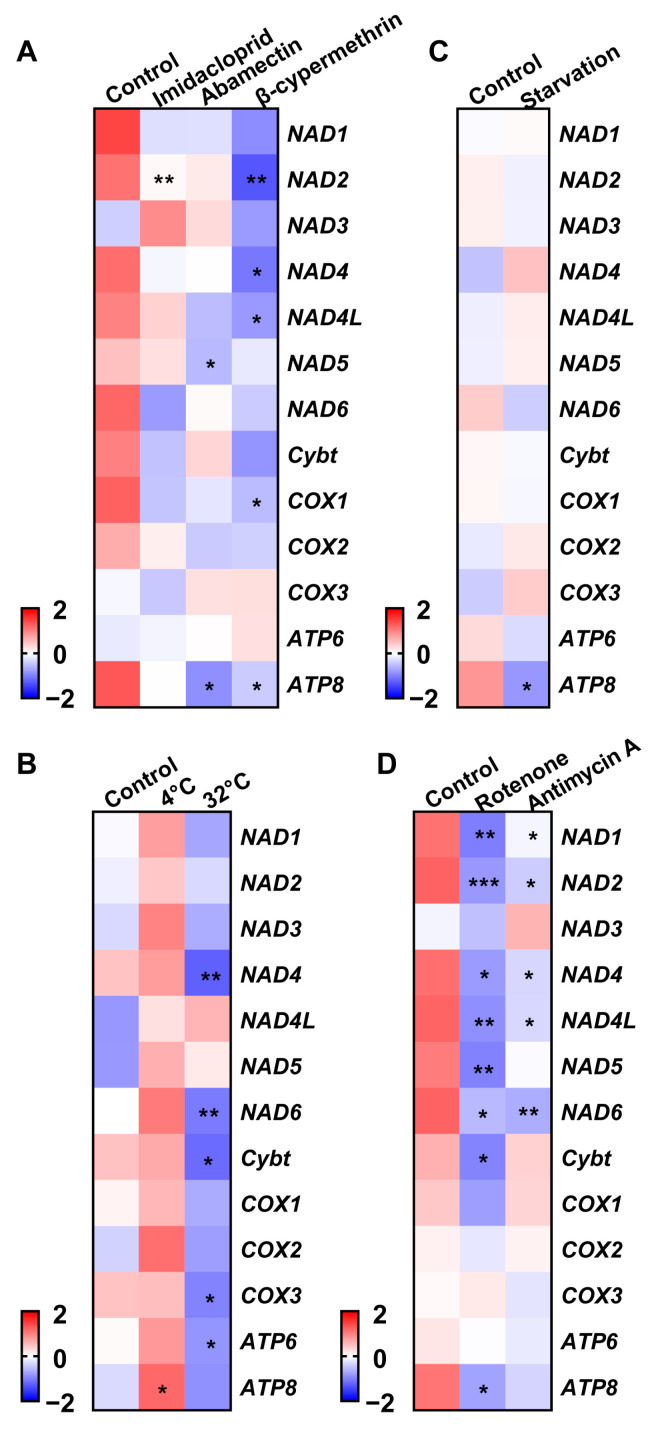
Transcriptional patterns of mitochondrial protein-coding genes in Acyrthosiphon pisum under different treatments. (**A**) Insecticide exposure. (**B**) Varying temperatures. (**C**) Starvation. (**D**) Mitochondrial inhibitors. Values represent the mean ± standard error (SE) of three biological replicates. Asterisks denote statistically significant differences as determined by Student’s *t*-test (* *p* < 0.05, ** *p* < 0.01, *** *p* < 0.001).

Treatment-induced changes included imidacloprid downregulating NAD2; abamectin reducing NAD5 and ATP8; and β-cypermethrin suppressing NAD2, NAD4, NAD4L, COX1, and ATP8 (Figure 4A). Low temperatures (4 °C) upregulated ATP8, while high temperatures decreased NAD4, NAD6, Cytb, COX3, and ATP6 (Figure 4B). Starvation specifically downregulated ATP8 (Figure 4C). Rotenone reduced NAD1, NAD2, NAD4, NAD4L, NAD5, NAD6, Cytb, and ATP8, whereas antimycin A suppressed NAD1, NAD2, NAD4, NAD4L, NAD6, and ATP8 (Figure 4D).

**Figure 5:**
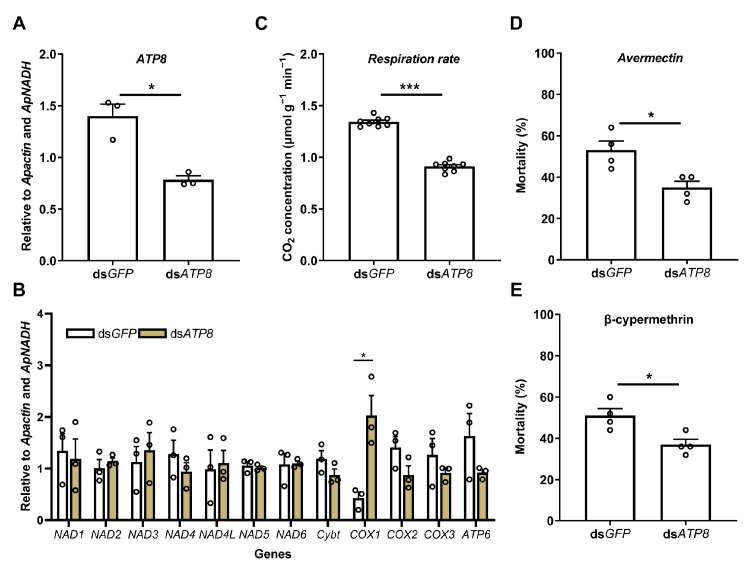
Respiration rates of *Acyrthosiphon pisum* under the silencing of a key mitochondrial protein-coding gene (*ATP8*), using the portable photosynthesis-monitoring system. Effects of ATP8 silencing on the respiration rates and stress resistance of *Acyrthosiphon pisum*. (**A**) Relative expression level of *ATP8* after *ATP8* dsRNA treatment. (**B**) Relative expression levels of other mitochondrial protein-coding genes after *ATP8* dsRNA treatment. (**C**) Respiration rates of *Ac. pisum* after *ATP8* dsRNA treatment. (**D**) Mortality of *Ac. pisum* under avermectin treatment upon feeding dsRNA. (**E**) Mortality of *Ac. pisum* under β-cypermethrin treatment upon feeding dsRNA. Values represent the mean ± standard error (SE) of three biological replicates for gene expression levels, eight for respiration rate, and four for mortality. Asterisks denote statistically significant differences as determined by Student’s *t*-test (* *p* < 0.05, *** *p* < 0.001).

Given the downregulation of *ATP8* under multiple stresses (abamectin, β-cypermethrin, starvation, and rotenone), this gene was further used to measure the respiration rates of *Ac. pisum* under the silencing of a key mitochondrial protein-coding gene, using the portable photosynthesis-monitoring system. The successful silencing of ATP8 (44.1% efficiency) (Figure 5A) led to significant changes in other mitochondrial genes, with COX1 being significantly upregulated (Figure 5B). The silenced-gene insects exhibited significantly lower respiration rates compared to the dsGFP controls (0.91 vs. 1.34 μmol g^−1^ min^−1^, Figure 5C). Additionally, the dsATP8-treated groups demonstrated enhanced resistance to abamectin (a 15.2% lower mortality rate, Figure 5D) and β-cypermethrin (a 13.6% lower mortality rate, Figure 5E).

## 4. Discussion

In this study, we demonstrated the feasibility of using a portable photosynthesis system to measure respiration rates in small-sized insects, providing a practical and efficient approach for field-based studies. The results revealed significant variation in respiration rates across developmental stages and species, which aligns with previous reports showing that metabolic demands increase with higher energy requirements for activities such as flight, reproduction, stress resistance, and foraging [7,42]. A prior study indicated that geographic populations of three beetle species from different regions of Brazil exhibited significant differences in respiration rates [14]. Similarly, in *Helicoverpa armigera*, respiration rates fluctuated dynamically across developmental stages, peaking temporarily during interlarval molting and metamorphosis to pupae, sharply declining in the pupal phase, and rising again immediately before eclosion [15]. The high sensitivity and reproducibility rates of the portable photosynthesis system establish it as a valuable tool for future research, particularly in ecological and environmental studies requiring field measurements. Going forward, this system could be used to measure the respiration rates of extremely small insects and assess their stabilities under extreme environmental temperature and humidity conditions to test the detection limits of the system.

The respiration rates of *Ac. pisum* were differentially influenced by environmental and chemical treatments. Light/dark cycles showed minimal effects on metabolic activity, suggesting stable respiratory regulation under variable light conditions. In contrast, significant changes were observed under insecticide exposure, with all the compounds reducing respiration rates, likely due to mitochondrial disruption. High temperatures increased respiration rates, while lower temperatures suppressed them. Starvation similarly reduced respiration, consistent with energy conservation strategies during nutrient deprivation. Mitochondrial inhibitors further confirmed respiratory suppression through targeted interference. These patterns align with observations in other insects. For example, starvation reduced respiration in *H. armigera* [15], and mitochondrial inhibitors (rotenone, malonate, and antimycin A) decreased respiratory activity in the flight muscles of *Vespula vulgaris*, *Bombus impatiens*, *Apis mellifera*, and *Locusta migratoria* [16]. Similar effects were reported for imidacloprid in *Euschistus heros* males [43], plant-derived compounds (lemongrass essential oil, geranyl acetate, cinnamon terpenoids, clove oils, and citral) in *Sitophilus granarius* and *Ulomoides dermestoides* [44,45,46], squamocin/tebufenozide in *Anticarsia gemmatalis* larvae [47,48], *Cymbopogon citratus* essential oil in *Podisus nigrispinus* [7], and chlorantraniliprole in *Hypothenemus hampei* [17], collectively demonstrating the broad conservation of metabolic responses across taxa.

Mitochondrial gene expression dynamics reveal key regulatory mechanisms underlying respiratory plasticity during development and in response to environmental stress. Under light–dark cycles, most mitochondrial genes exhibit stable expression levels, with small fluctuations observed at the end of light phases or during periods of darkness. Analyses across developmental stages revealed high expression levels of *NAD1*, *NAD4*, *NAD5*, *Cytb*, *COX1-3*, and *ATP6* in early nymphal stages, reflecting the increased energy demands for growth. Lower expression levels in later nymphs and adults suggest a shift in energy use toward maintenance and reproduction, which aligns with observations of low *ATG5* levels during early diapause, followed by peak expression after 12 weeks of diapause induction [49]. Cold stress was shown to increase *ATP8* expression, likely as a response to maintain energy balance through adaptive processes, while starvation led to a decrease in *ATP8* expression to conserve resources. Additionally, mitochondrial inhibitors generally reduced the expression levels of electron-transport-chain (ETC) genes, indicating the direct suppression of mitochondrial function. These results corroborate those of earlier studies. For example, lower expression levels of *Cytb*, *NAD3*, *NAD5*, *NAD6*, *ATP6*, and *ATP8* were observed in *Cryptolestes ferrugineus* resistant to phosphine [37], contrasting with the higher expression levels of *COX1-3*, *NAD1*, *NAD4*, *NAD5*, *ATP6*, and *Cytb* in *Monochamus alternatus* during pinewood nematode infection [36]. Furthermore, imidacloprid exposure reduces the expression levels of *COX3*, *NAD4*, and *NAD4L* in *Choroterpes yixingensis* [9], while long-term cold stress lowered the expression levels of *NAD1*, *NAD4*, *NAD4L*, *NAD5*, *COX1*, *COX3*, *ATP6*, and *ATP8* in *C. ferrugineus* [9]. These findings suggest both shared and distinct effects of chemical and cold stress on mitochondrial gene networks. Additionally, pyrethroid-resistant *Anopheles sinensis* populations exhibited higher *NAD5* expression and lower *ATP8* expression levels [50], further highlighting the evolutionary conservation of mitochondrial regulatory systems that enable adaptation to different stressors.

RNAi-targeting *ATP8* provided functional evidence for its role in regulating respiration. The silencing of *ATP8* significantly reduced respiration rates, indicating its critical role in maintaining aerobic energy production. Notably, the compensatory upregulation of *COX1* was observed, suggesting the potential mitochondrial complementary mechanisms that help to stabilize respiration rates and energy homeostasis. Interestingly, the ds*ATP8*-treated insects exhibited lower respiration rates and reduced mortality rates under exposure to abamectin and β-cypermethrin, implying that *ATP8* downregulation may reduce metabolic rates, thereby decreasing toxin uptake and insecticide susceptibility. This protective effect could stem from optimized energy allocation to counteract chemical toxicity. Similar patterns have been observed in other mitochondrial genes. For instance, *NAD6* knockdown resulted in increased mortality rates, whereas the suppression of *COX1* reduced mortality rates following exposure to allyl isothiocyanate [51]. Similarly, *NAD4* silencing led to the broad downregulation of mitochondrial genes (including *NAD1*, *NAD2*, *NAD4L*, *COX1*, *COX3*, *ATP6*, and *ATP8*) in a manner similar to that of the transcriptional cascade triggered by *COX3* knockdown, which further reduced respiration rates but improved cold tolerance in *C. ferrugineus* [9]. Studies on *B. dorsalis* revealed that avermectin and malathion induced *COX2* expression, and silencing *COX2* increased pesticide-induced mortality rates [11]. These findings align with the idea that metabolic suppression may be linked to toxin tolerance. Reduced energy flux could limit toxin uptake or activate survival pathways, as seen in phosphine-resistant insects and nematodes with impaired mitochondrial function [52,53].

## 5. Conclusions

In conclusion, the use of a portable photosynthesis system for measuring respiration rates in small-sized insects opens up new possibilities for field-based studies, enabling a better understanding of how climate change and pest control measures affect insect population dynamics. Furthermore, the comprehensive analysis of mitochondrial gene expression, coupled with functional validation through RNAi, not only enhances our understanding of the molecular mechanisms governing respiration but also identifies potential targets for the development of more effective and environmentally friendly pest control strategies.

## Data Availability

The original contributions presented in this study are included in the article/Supplementary Material. Further inquiries can be directed to the corresponding author.

## Supplementary Materials

The following supporting information can be downloaded at https://www.mdpi.com/article/10.3390/insects16060616/s1: Table S1: Primers used in this study. Figure S1: Transcriptional patterns of mitochondrial protein-coding genes in *Acyrthosiphon pisum* during light/dark periods. Figure S2. Transcriptional patterns of mitochondrial protein-coding genes in *Acyrthosiphon pisum* during different developmental stages.
