# Supplementary material for: Development of an Experimental Method Using a Portable Photosynthesis-Monitoring System to Measure Respiration Rates in Small-Sized Insects
Source: Insects. 2025 Jun 10;16(6):616. doi: 10.3390/insects16060616 (PMC12193473; doi:10.3390/insects16060616)
Supplement: Supplementary file 1 [file insects-16-00616-s001.zip › insects-3624055-supplementary/Table S1.pdf]

Table S1. Primers used in this study.

genes	Forward（5’-3’）	Reverse（5’-3’）	E
Cox1	CACGAGCATATTTCACATCAGCA	ACACCTGTTAACCCACCGAT	106.8%
Cox2	ATTGATGCTATTCCAGGACGTA	CCACAAATTTCTGAGCATTGACC	96.4%
Cox3	AGCAACATTTACATTCTCAGATTCA	ACGTGTAATCCATGAAATCCTGT	107.7%
Atp6	GCACATTTAATTCCACTTAATACACCA	GTCGAATTGATAATGATAAAGGTCGGA	102.9%
Atp8	ATAGCACCAATTAATTGATTAATTTTATTT	TTAAATAAATTTATTATAATTTTTTGTATTGTTTAT	87.5%
Nad1	TGGTAAAAATCCACGTAATCAAATAAC	TCTCCTTTTGATTTTTCTGAAGGTGA	97.9%
Nad2	CCATTTCATTTATGATTACCTTCTAT	TCATGTTGTGTTATTGATTGAAGA	94.0%
Nad3	CCATTTAATAAATCACGAATTCCTTTCTC	TTAGATTTTTCAATTTAGTGATCCAAATT	93.8%
Nad4	TCACCAAATAAATTCAAAGAAGGAGGA	TGGTCATGGTTTATGTTCTTCTGG	111.7%
Nad4l	AAACCTAATACTCTTTCACTAATACAAAA	GTTGTAATATTTATGTTATTTTCTGGAGT	106.6%
Nad5	AGAAACAGGAGTAGGAGCTGC	TCTTATAGTTCAGGATTGGTTACTGT	113.0%
Nad6	TTTAACAATAATAAAATCACCTATTAGATC	GAATAAAAATTATAAATGAAATTCATGCTG	104.3%
Cytb	TGTAGGATATGTACTACCTTGAGGA	ATGGAATTGCTGAAAGAAGATTTGT	99.1%
Apactin	CGAGGAGAACATGCTCTTAGAC	GATAGCTTGGGCTGGACATATAG	109.0%
ApNADH	CGTTACCAACTGGGACGATATG	GGGTTCAATGGAGCTTCTGTTA	106.2%
