# Supplementary figures and images for: Development of an Experimental Method Using a Portable Photosynthesis-Monitoring System to Measure Respiration Rates in Small-Sized Insects
Source: Insects. 2025 Jun 10;16(6):616. doi: 10.3390/insects16060616 (PMC12193473; doi:10.3390/insects16060616)

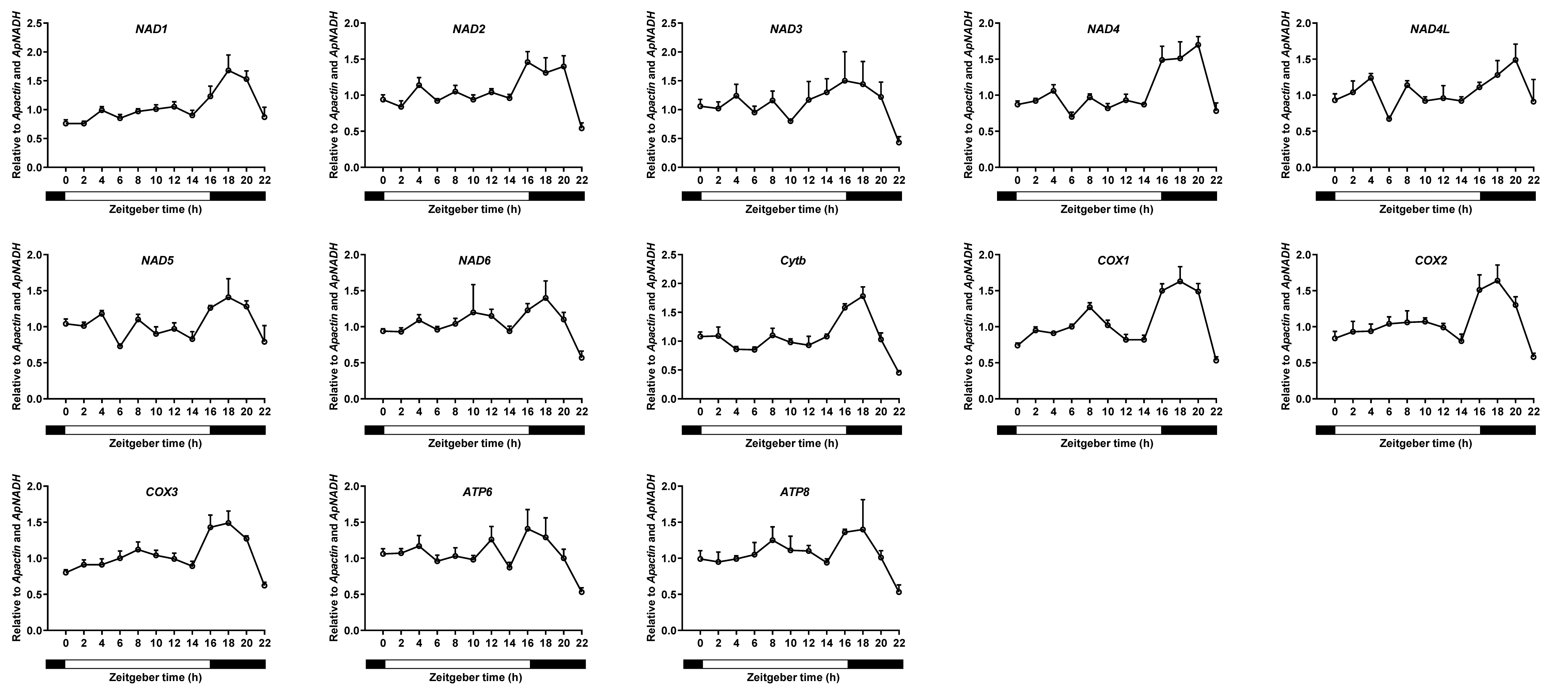

Supplement: Supplementary file 1 [file insects-16-00616-s001.zip › insects-3624055-supplementary/Figure S1.tif]
